# The Group Decision-Making Using Pythagorean Fuzzy Entropy and the Complex Proportional Assessment

**DOI:** 10.3390/s22134879

**Published:** 2022-06-28

**Authors:** Parul Thakur, Bartłomiej Kizielewicz, Neeraj Gandotra, Andrii Shekhovtsov, Namita Saini, Wojciech Sałabun

**Affiliations:** 1 Yogananda School of AI, Computer and Data Sciences, Faculty of Engineering and Technology, Shoolini University, Solan 173229, Himachal Pradesh, India; parult147@gmail.com (P.T.); neerajgandotra@shooliniuniversity.com (N.G.); namita@shooliniuniversity.com (N.S.); 2 Research Team on Intelligent Decision Support Systems, Department of Artificial Intelligence and Applied Mathematics, Faculty of Computer Science and Information Technology, West Pomeranian University of Technology in Szczecin, ul. Żołnierska 49, 71-210 Szczecin, Poland; bartlomiej-kizielewicz@zut.edu.pl (B.K.); andrii-shekhovtsov@zut.edu.pl (A.S.); 3 National Institute of Telecommunications, Szachowa 1, 04-894 Warsaw, Poland

**Keywords:** entropy, pythagorean fuzzy sets, multiple criteria decision analysis, decision-making, complex proportional assessment

## Abstract

The Pythagorean fuzzy sets conveniently capture unreliable, ambiguous, and uncertain information, especially in problems involving multiple and opposing criteria. Pythagorean fuzzy sets are one of the popular generalizations of the intuitionistic fuzzy sets. They are instrumental in expressing and managing hesitant under uncertain environments, so they have been involved extensively in a diversity of scientific fields. This paper proposes a new Pythagorean entropy for Multi-Criteria Decision-Analysis (MCDA) problems. The entropy measures the fuzziness of two fuzzy sets and has an influential position in fuzzy functions. The more comprehensive the entropy, the more inadequate the ambiguity, so the decision-making established on entropy is beneficial. The COmplex PRoportional ASsessment (COPRAS) method is used to tackle uncertainty issues in MCDA and considers the singularity of one alternative over the rest of them. This can be enforced to maximize and minimize relevant criteria in an assessment where multiple opposing criteria are considered. Using the Pythagorean sets, we represent a decisional problem solution by using the COPRAS approach and the new Entropy measure.

## 1. Introduction

Decision-making methods reflect how decisions are completed and how they can be completed better or more successfully. The decision-making process can also be described as evaluating successive decision alternatives and is broadly used in both the social sciences and the more demanding natural sciences and engineering. The fuzzy sets generalizations are involved in the process of decision making by modeling the value of decisional attributes with uncertainty [1]. A very important aspect is that, unlike probabilistic theories and game theory, these generalizations are used to handle incomplete and uncertain information formulated by the decision-maker [2].

When we have complete and accurate information about each decision attribute and know the crisp values of the decision criteria weights, we deal with decision-making with certainty. On the other hand, when the information is uncertain, the values are unknown or given in some approximation, then we are dealing with decision-making under uncertainty. One approach successfully used to model uncertainty is fuzzy set theory. A fuzzy set is often a functional relationship linking a feature value to a so-called membership function. A domain expert often identifies this function and less often by physical readings. Fuzzy sets as a generalization of classical two-state logic allow better reality modeling, especially in an uncertain environment [3,4]. Fuzzy sets have been widely used in technics and complex Multi-Criteria Decision-Analysis (MCDA) problems. They have been prevalent since they were proposed by Zadeh [5].

Considerable mathematicians put forth exceptional tries to compute the concept of fuzzy sets in various substantial manners. To overcome the confinements of the fuzzy set in managing conflicting statements regarding the enrollment of articles and decision-making, in 1986, Atanassov submitted the intuitionistic fuzzy set [6,7]. The intuitionistic fuzzy sets are progressively effective and valuable because they execute the extents of conviction, incredulity, and hesitation margin simultaneously [8]. An intuitionistic fuzzy set is portrayed by two functions, i.e., a membership and a non-membership function. They specify the degree of membership and non-membership of each element [9]. The degree of hesitancy is 1 minus the degrees of membership and non-membership. Thus, the aggregate of both significances is smaller than one [3]. With the two functions relating to membership and non-membership and the hesitancy index of an Intuitionistic Fuzzy Sets (IFS), we can express three states: support state, opponent state, and neutral state [10].

An intuitionistic fuzzy set is a powerful tool for dealing with indeterminate or uncertain information in decision making, mathematical programming tasks, medical diagnosis cases [11], pattern recognition problems [12], and human expressions such as behavior, perception, and understanding [13]. Many researchers have successfully used intuitionistic fuzzy theory in decision-making at different stages of this process [14,15,16,17]. The concept of multi-criteria decision analysis (MCDA) involves using expert knowledge. The expert’s task is to evaluate different alternatives concerning specific criteria.

One way of expressing expert knowledge in MCDA methods is a characteristic objects comparison approach called Characteristic Object METhod (COMET) [18,19]. However, many popular multi-criteria decision analysis methods use expert knowledge in the form of weights to evaluate alternatives. Popular MCDA methods using this approach include Technique for the Order of Prioritisation by Similarity to Ideal Solution (TOPSIS) [20,21], VIseKriterijumska Optimizacija I Kompromisno Resenje (VIKOR) [22,23], Preference Ranking Organization METHod for Enrichment of Evaluations I & II (PROMETHEE) [24,25], Stable Preference Ordering Towards Ideal Solution (SPOTIS) [26,27], COmbined COmpromise SOlution (COCOSO) [28,29], Multi-Attributive Border Approximation area Comparison (MABAC) [30,31], or Additive Ratio ASsessment (ARAS) [32].

These weights can be assigned arbitrarily by an expert or using a subjective or objective method. In the case of objective weight assignment, the significance of the criteria is examined using a measure such as Entropy or Standard Deviation. In the subjective approach, a domain expert determines the significance of the criteria. The significance of criteria in subjective methods can be expressed by criterion rankings or pairwise criteria comparisons. Subjective weighting methods based on criterion rankings are the Best-Worst Method (BWM) [33], FUll COnsistency Method (FUCOM) [34], or Step-wise Weighting Assessment Ratio Analysis (SWARA) [35]. The most popular approach is the Analytical Hierarchy Process (AHP) regarding subjective weighting by pairwise comparisons [36].

Although these methods work on crisp values, they cannot be applied to matrices represented using fuzzy logic and extensions. Therefore, new extensions to these methods are proposed to account for uncertain data. Ghorabaee et al. proposed an extension of the SWARA and CRiteria Importance Through Intercriteria Correlation (CRITIC) approach to determine the criterion weights for a matrix represented using fuzzy sets [37]. Lyu et al. proposed an extension of the FAHP method with trapezoidal fuzzy numbers for evaluating urban infrastructure [38]. Finally, Joshi et al. presented a new decision-making method based on correlation coefficients that use entropy weights in an IFS environment [39].

The use of IFS has found wide application in real-life situations due to the excellent modeling properties of human knowledge in decision-making tasks such as public administration, engineering, management science, economics, military, and scientific research.

In some real-world problems solved by MCDA methods, it turns out that all the degrees of estimation of the membership and non-membership of the individual alternatives provided by the decision-maker can be determined such that their sum is greater than 1. Such a situation is inappropriate for applying intuitionistic fuzzy sets and makes uncertainty modeling difficult. Therefore, as an extension of intuitionistic fuzzy sets, a Pythagorean fuzzy set (PFS) is presented, which is more flexible in expressing and dealing with uncertainty and imprecision than intuitionistic fuzzy sets in various decision-making procedures.

Pythagorean fuzzy sets were initially developed by Atanassov in 1999 using the name “intuitionistic fuzzy sets of the second kind”. Yager presented the first real applications of PFS in decision-making in 2013 [40]. Yang and Hussain [41], and Zhang [42] proposed various new estimations of fuzzy entropy values for Pythagorean fuzzy sets depending on the probabilistic type, distance, and Pythagorean index.

MCDA provides decision-makers and experts with a wide range of methodologies well suited to the complexity of decision problems. MCDA methods mostly incorporate human participation and judgment. Pythagorean fuzzy sets provide better space for experts to depict their views concerning the vagueness and uncertainty of the considered Multi-Criteria Decision Making (MCDM) issues [43]. Since the introduction of Pythagorean fuzzy MCDA, researchers have shown great interest and concentration in exploring various applications of the Pythagorean fuzzy set.

Previous researchers have previously developed multiple MCDA approaches to illustrate the complex choice problems that arise in our daily life. Each choice problem consists of a set of alternatives, criteria, and a vector of weights and types of each criterion (cost or profit). The COPRAS method has been repeatedly applied to uncertainty problems, where uncertainty has been modeled mainly by fuzzy sets. This approach is a suitable and uncomplicated technique to succeed in MCDM problems. The COPRAS approach has been prolonged by countless scholars from the characteristics of composition in the most modern times. This approach estimates the individuality of one alternative over another and is an example of a reasonable equating of alternatives. This approach can maximize and minimize criteria in a study where more than one measure must be evaluated [44]. The COPRAS approach progressively ranks and evaluates alternatives in terms of their suitability and degree of use [45]. It is a progressive approach to solving real-world difficulties. Previous research has also combined the COPRAS method with the Pythagorean Fuzzy Sets environment. For example, Buyukozkan and Gocer used PFS COPRAS to evaluate alternative Digital Supply Chain partners [46]. In addition, Alipour et al. used the SWARA-COPRAS approach to select a fuel cell combined with hydrogen supplier [47].

Due to the vast pool of available tools related to multi-criteria decision analysis, the COPRAS approach has been compared more than once. For example, Özcan and Çelik compared the COPRAS method with the TOPSIS method in a machine selection problem in the food industry in Turkey [48]. Pamucar and et al. compared the COPRAS method with four other multi-criteria decision analysis methods in a site selection problem for developing a multimodal logistics center on the Danube River [49]. Borkar and et al. compared the COPRAS and ELECTRE approaches in the manufacturing domain problem [50]. Mulliner and et al. compared the WPM, WSM, and TOPSIS methods with the COPRAS technique in the sustainable housing affordability assessment problem [51].

### 1.1. Challenges and Motivation

Based on the above review, it can be concluded that the COPRAS method is a promising approach to decision support that has been developed relatively recently. Unfortunately, applications using Pythagorean Fuzzy Sets are pretty limited. Therefore, further work should be conducted towards developing the COPRAS method using entropy measures. The entropy measure in PFS is also an interesting issue, as it is the basis for determining weights in decision matrices in the COPRAS technique. Research towards group decision-making is directed toward aggregating expert knowledge, which is one of the main challenges of group decision-making. Many techniques related to multi-criteria decision analysis provide an answer for a single subjective set of evaluations, so they are extended to the topic of group decision-making. In addition, there is a difficulty associated with incorporating uncertain data into the process of expert knowledge extraction.

Due to the above limitations associated with group decision-making, the motivation of this paper is as follows:

- Some works have omitted research on the sensitivity analysis of the COPRAS method in a PFS environment [52];
- Several papers miss the process of assigning weights in the aggregate preference matrix of Pythagorean Fuzzy Sets decision-makers [53];
- Sensitivity analysis of the proposed entropy methods in Pythagorean Fuzzy Sets is missing;
- The small number of approaches related to entropy determination in the Pythagorean Fuzzy Sets environment results in a lack of comparisons and accurate statements.

### 1.2. Contribution and Novelties

This paper focuses on using the Pythagorean Fuzzy COPRAS approach in group decision-making. A new Pythagorean Fuzzy Entropy measure is proposed to determine the attribute weights for the group decision-making problem. The proposed approach is applied to a crop field evaluation problem involving four domain experts. In addition, a sensitivity analysis is performed in this paper to check the accuracy of the proposed approach. The main contribution of our work is applying the new Pythagorean Fuzzy Entropy measure combined with the COPRAS method for crop field evaluation.

### 1.3. Framework of This Study

The rest of the paper is organized as follows. Section 2 gives a brief introduction to the Pythagorean fuzzy set. In the next Section 3, a new entropy measure PFS is proposed. Section 4 presents a practical application in decision-making and sensitivity analysis of the proposed approach. Finally, Section 5 concludes with a summary of the paper and directions for future work.

## 2. Preliminaries

Yager [54] defines the Pythagorean fuzzy set as: Let us consider a non-empty and finite set *N* of nominal elements, $N=\left\{n_{1},\;n_{2},\ldots ,n_{m}\right\}$. A Pythagorean fuzzy set *K* in the crisp set *N* may be represented as (1):

(1) $$K=\left\{\left(n,\;\mu_{K}\left(n\right),\;\vartheta_{K}\left(n\right)\right)|n\in N\right\}$$

where $\mu_{K}\left(n\right):N\to \left[0,\;1\right]$ and $\vartheta_{K}\left(n\right):N\to \left[0,\;1\right]$ with the condition $0\leq {\mu_{K}}^{2}\left(n\right)+{\vartheta_{K}}^{2}\left(n\right)\leq 1$.

The $\mu_{K}\left(n\right)$ is the degree of membership of element n∈N; $\vartheta_{K}\left(n\right)$ is the degree of non-membership of the element n∈N.

## 3. The New Entropy for Pythagorean Fuzzy Set

The fuzzy set theory causes the use of entropy to estimate the degree of fuzziness in a fuzzy set. Fuzzy entropy describes the mathematical importance of the fuzziness of fuzzy sets. Classical Shannon entropy is involved with probabilistic uncertainties, whereas fuzzy entropy is involved with randomness, vagueness, fuzziness, and ambiguous uncertainties [55]. Axiomatic entropy of fuzzy sets is persisted as the Pythagorean fuzzy sets [56]. The entropy of fuzzy sets measures the fuzziness of two fuzzy sets. It has an influential place in fuzzy functions, such as fuzzy neural network functions, fuzzy knowledge base functions, fuzzy decision-making functions, fuzzy management functions, and fuzzy management details functions [57].

**Definition** **1.** *A real function E: pythagorean fuzzy set $\left(N\right)\to \left[0,\;1\right]$ is characterized as entropy on pythagorean fuzzy set (N) if E satisfies the latter axioms [58]:*

- ***Minimality:*** *$E\left(K\right)=0,$ if A is crisp set;*
- ***Maximality:*** *$E\left(K\right)=1,$ if $\mu_{K}\left(n\right)=\vartheta_{K}\left(n\right)=\frac{1}{\sqrt{3}}\;,\;\forall n;$*
- ***Resolution:*** *$E\left(K\right)\leq E\left(L\right),$ if K is less fuzzy than L, i.e., $\mu_{K}\left(n\right)\leq \mu_{L}\left(n\right)\leq \frac{1}{\sqrt{3}}$ and $\vartheta_{L}\left(n\right)\leq \vartheta_{K}\left(n\right)\leq \frac{1}{\sqrt{3}}$ for $\mu_{L}\left(n\right)\leq \mu_{K}\left(n\right)$ or $\mu_{K}\left(n\right)\geq \mu_{L}\left(n\right)\geq \frac{1}{\sqrt{3}}$ and $\vartheta_{L}\left(n\right)\geq \vartheta_{K}\left(n\right)\geq \frac{1}{\sqrt{3}}$ for $\mu_{L}\left(n\right)\geq \mu_{K}\left(n\right);$*
- ***Symmetry:*** *$E\left(K\right)=E\left(K^{c}\right),$ where $K^{c}$ is the complement of K.*

*According to Pythagorean fuzzy knowledge, we propose that this last Pythagorean fuzzy entropy is analogous to the criteria:*

*Let $N=n_{1},n_{2},n_{3},\cdots ,n_{m}$ be the universal set. Let $A=\left\{\left(n_{i},\;\mu_{a}\left(n_{i}\right),\;\vartheta_{a}\left(n_{i}\right)|n_{i}\in N\right)\right\}$ be a pythagorean fuzzy set on N.*

(2) $$E_{p}\left(A\right)=\frac{1}{m}\sum_{i=1}^{m}E_{p}^{\#}\left(S\right)$$

*where,*

(3) $$\begin{array}{cc} E_{p}^{\#}\left(A\right)= & \frac{1}{1+\sqrt{e}}\left[\log\left(2-\mu_{a}\left(n_{i}\right)+\vartheta_{a}\left(n_{i}\right)\right)+\log\left(2+\mu_{a}\left(n_{i}\right)-\vartheta_{a}\left(n_{i}\right)\right)-\sqrt{0.22764}\right]\times \\ \; & \times \left(\frac{1}{0.04717}\right) \end{array}$$

*for all i = 1, 2, 3, …, m.*

**Theorem** **1.** *The measure $E_{p}\left(A\right)$ is a valid entropy.*

**Proof.**  To prove the proposed measure is valid, we have to show that it satisfies the properties as provided in Definition 1.

1. Minimality: if *A* is a crisp set, i.e., $\mu_{a}\left(n_{i}\right)=1,\;\vartheta_{a}\left(n_{i}\right)=0$ or $\mu_{a}\left(n_{i}\right)=0,\;\vartheta_{a}\left(n_{i}\right)=1$ for all $n_{i}\in N$ then,
  $$\frac{1}{1+\sqrt{e}}\left[\log\left(2-\mu_{a}\left(n_{i}\right)+\vartheta_{a}\left(n_{i}\right)\right)+\log\left(2+\mu_{a}\left(n_{i}\right)-\vartheta_{a}\left(n_{i}\right)\right)-\sqrt{0.22764}\right]\times \left(\frac{1}{0.04717}\right)=0$$
  Therefore, $E_{p}\left(A\right)=0$
2. Maximility: for all $n_{i}\in N$, if $\mu_{a}\left(n_{i}\right)=\vartheta_{a}\left(n_{i}\right)=\frac{1}{\sqrt{3}}$ then, $\frac{1}{1+\sqrt{e}}\left[\log\left(2-\mu_{a}\left(n_{i}\right)+\vartheta_{a}\left(n_{i}\right)\right)\right.$ $+\left.\log\left(2+\mu_{a}\left(n_{i}\right)-\vartheta_{a}\left(n_{i}\right)\right)-\sqrt{0.22764}\right]\times \left(\frac{1}{0.04717}\right)=1,$ for all $n_{i}\in N$.
3. Resolution: in order to prove the fourth property, consider the function $f\left(\mu ,\;\vartheta \right)$ such that (4):
  (4) $$\begin{array}{cc} f\left(\mu ,\;\vartheta \right)= & \frac{1}{1+\sqrt{e}}\left[\log\left(2-\mu_{a}\left(n_{i}\right)+\vartheta_{a}\left(n_{i}\right)\right)+\log\left(2+\mu_{a}\left(n_{i}\right)-\vartheta_{a}\left(n_{i}\right)\right)-\right.\\ \; & \left.-\sqrt{0.22764}\right]\times \left(\frac{1}{0.04717}\right) \end{array}$$
  where μ,ϑ∈[0,1].
  The partial derivatives with respect to μ (5) and ϑ (6) are obtained as,
  (5) $$\frac{\partial f}{\partial \mu }=\frac{1}{1+\sqrt{e}}\left[\frac{-1}{2-\mu +\vartheta }+\frac{1}{2+\mu -\vartheta }\right]\times \left(\frac{1}{0.04717}\right)$$
  (6) $$\frac{\partial f}{\partial \vartheta }=\frac{1}{1+\sqrt{e}}\left[\frac{1}{2-\mu +\vartheta }+\frac{-1}{2+\mu -\vartheta }\right]\times \left(\frac{1}{0.04717}\right)$$
  We obtained that, $\frac{\partial f\left(\mu ,\;\vartheta \right)}{\partial \mu }\geq 0$ when μ≤ϑ and $\frac{\partial f\left(\mu ,\;\vartheta \right)}{\partial \mu }\leq 0$ when μ≥ϑ, whereas; $\frac{\partial f\left(\mu ,\;\vartheta \right)}{\partial \vartheta }\leq 0$ when μ≤ϑ and $\frac{\partial f\left(\mu ,\;\vartheta \right)}{\partial \vartheta }\geq 0$ when μ≥ϑ. Thus *f* is increasing with respect to *μ* when μ≤ϑ and decreasing when μ≥ϑ. Moreover, *f* is decreasing with respect to ϑ when μ≤ϑ and increasing when μ≥ϑ.
  Presently, by using this property of the function, we can conclude that $E\left(A\right)\leq E\left(\dot{A}\right)$, if *K* is less fuzzy than *L*, i.e., $\mu_{K}\left(n\right)\leq \mu_{L}\left(n\right)\leq \frac{1}{\sqrt{3}}$ and $\vartheta_{L}\left(n\right)\leq \vartheta_{K}\left(n\right)\leq \frac{1}{\sqrt{3}}$ for $\mu_{L}\left(n\right)\leq \mu_{K}\left(n\right)$ or $\mu_{K}\left(n\right)\geq \mu_{L}\left(n\right)\geq \frac{1}{\sqrt{3}}$ and $\vartheta_{L}\left(n\right)\geq \vartheta_{K}\left(n\right)\geq \frac{1}{\sqrt{3}}$ for $\mu_{L}\left(n\right)\geq \mu_{K}\left(n\right).$
4. Symmetry: for the property we have $A=\left(\mu_{a},\;\vartheta_{a}\right)$ as $A^{c}=\left(\vartheta_{a},\;\mu_{a}\right)$. Thus, we have (7),
  (7) $$\begin{array}{cc} E_{p}^{\#}\left(A^{c}\right)= & \frac{1}{1+\sqrt{e}}\left[\log\left(2-\vartheta_{a}\left(n_{i}\right)+\mu_{a}\left(n_{i}\right)\right)+\log\left(2+\vartheta_{a}\left(n_{i}\right)-\mu_{a}\left(n_{i}\right)\right)-\sqrt{0.22764}\right]\times \left(\frac{1}{0.04717}\right)=\\ = & \frac{1}{1+\sqrt{e}}\left[\log\left(2-\mu_{a}\left(n_{i}\right)+\vartheta_{a}\left(n_{i}\right)\right)+\log\left(2+\mu_{a}\left(n_{i}\right)-\vartheta_{a}\left(n_{i}\right)\right)-\sqrt{0.22764}\right]\times \left(\frac{1}{0.04717}\right)=\\ = & E_{p}^{\#}\left(A\right) \end{array}$$

Hence, $E_{p}^{\#}\left(A^{c}\right)=E_{p}^{\#}\left(A\right)$.□

## 4. Application of the Proposed Entropy with the Copras Method

### 4.1. Study Case

**Figure 1:**
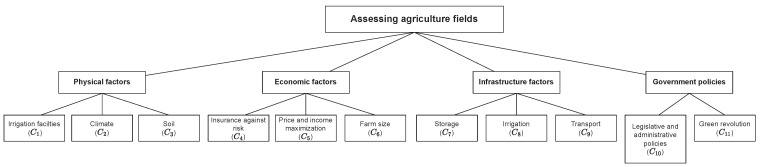
Framework of Evaluation Criteria.

In this section, we use the Pythagorean fuzzy entropy in the COPRAS approach for assessing five agriculture fields $\left(A_{1},A_{2},A_{3},A_{4},A_{5}\right)$. An agricultural scientist reviews for specific points, incorporating a short priority sheet for checking the agriculture field. The priority from an agricultural scientist (decision-maker) for a particular criterion has been taken in terms of linguistic variables – Excellent, Good, Average, Poor, and Very Poor are used in this research paper to determine the standard level. For a particular criterion, the agricultural scientist will indicate the degree of priority level on a discrete scale of 1 (Very Poor) to 5 (Excellent). The broad category of the priority sheet has been chosen to be a physical factor, economic factor, infrastructure facilities, and government policies. Further, in the priority sheet, these categories have been sub-divided into eleven different evaluation criteria from top to bottom in the framework shown in Figure 1. Four primary criteria classifications were considered, i.e., Physical, Economic, Infrastructure, and Government policies. The Physical factors included criteria mainly related to cultivation, such as irrigation facilities ($C_{1}$), climate ($C_{2}$), and solid ($C_{3}$). The economic factors included attributes related to the economics of maintaining agricultural fields, such as insurance against risk ($C_{4}$), price and income maximization ($C_{5}$), and farm size ($C_{6}$). For Infrastructure factors, storage ($C_{7}$), irrigation ($C_{8}$), and transport ($C_{9}$) were selected. Finally, for Government policies, the criteria were legislative and administrative policies ($C_{10}$) and the green revolution ($C_{11}$).

**Step I:** The decision-makers qualitatively expressed the linguistic evaluations for the 11 criteria under consideration (Table 1). They have been transformed into Pythagorean fuzzy information using their quantitative ratings in the Pythagorean fuzzy number (PFN) scale given in Table 2. We have a PFN value and a numeric symbol for each linguistic expression from 1 to 5. Moreover, the decision makers provide the qualitative information for five agriculture fields $A_{1},A_{2},A_{3},A_{4}$ and $A_{5}$ concerning criteria (Table 3) was transformed into Pythagorean fuzzy information by using the defined quantitative rating in PFNs scale given in Table 4.

**Step II:** In this step, we start by evaluating the importance of the decision makers using the previously defined linguistic terms, which are then transformed into fuzzy information using the Pythagorean approach with defined quantitative scores on the PFNs scale given in Table 2. We then calculate the weights of the decision makers using the given Equation (8):

(8) $$w_{i}=\frac{\mu_{a}^{2}\left(n_{i}\right)+\pi_{a}^{2}\left(n_{i}\right)\left[\frac{\mu_{a}^{2}\left(n_{i}\right)}{\mu_{a}^{2}\left(n_{i}\right)+\vartheta_{a}^{2}\left(n_{i}\right)}\right]}{\sum_{i=1}^{m}\mu_{a}^{2}\left(n_{i}\right)+\pi_{a}^{2}\left(n_{i}\right)\left[\frac{\mu_{a}^{2}\left(n_{i}\right)}{\mu_{a}^{2}\left(n_{i}\right)+\vartheta_{a}^{2}\left(n_{i}\right)}\right]}$$

where $\sum_{i=1}^{m}w=1$ and w≥0. The decision makers weights are given in the Table 5.

**Step III:** Presently, using the Pythagorean fuzzy weighted averaging aggregation operator given as (9):

(9) $$r_{ij}=\left(\sqrt{1-\prod_{i=1}^{m}{\left(1-\mu_{ij}^{2}\right)}^{w_{i}}},\;\prod_{i=1}^{m}{\left(\vartheta_{ij}\right)}^{w_{i}}\right)$$

The individual decision matrices received from different decision-makers are then aggregated into one main decision matrix. The resulting aggregated decision matrix is shown in Table 6 ($N_{pq}$).

**Step IV:** In this, we normalize the obtained aggregated pythagrean fuzzy deicion matrix using the given Equation (10):

(10) $$r_{\mathrm{ij}}=\left(\mu_{\mathrm{ij}},\vartheta_{\mathrm{ij}}\right)=\left\{\begin{array}{c} {\tilde{r}}_{\mathrm{ij}},\;\mathrm{for}\;\mathrm{benefits}\;\mathrm{criteria};\;\\ {\tilde{r}}_{\mathrm{ij}};\;,\;\mathrm{for}\;\cos t\;\mathrm{criteria}.\; \end{array}\right.$$

The normalized aggregated pythagorean fuzzy decision matrix is presented in Table 7 (${N^{\prime }}_{pq}$).

**Step V:** In this step, we will calculate the entropy for the given criteria using the proposed Pythagorean fuzzy entropy, shown in Table 8. Using the entropies next, we will calculate the weights given in Table 9.

**Step VI:** Determining the Criteria’s weight

Considering the vector containing the criteria weights has a decisive effect on the ranking order of the alternatives. In the proposed approach, we identify the vector of criteria weights using a newly developed entropy measure.

- For unknown criteria weights:
  If the criteria weights are completely unknown, then they are determined using the following Equation (11):
  (11) $$w_{j}=\frac{1-E_{p}\left(A\right)}{\sum_{i=1}^{m}\left(1-E_{p}\left(A\right)\right)},\;\left(t=1,\;2,\;\ldots ,m\right)$$
  where $E_{p}\left(A\right)=\frac{1}{m}\sum_{i=1}^{m}E_{p}^{\#}\left(A\right)$.

Criteria weights are shown in the Table 9.

**Figure 2:**
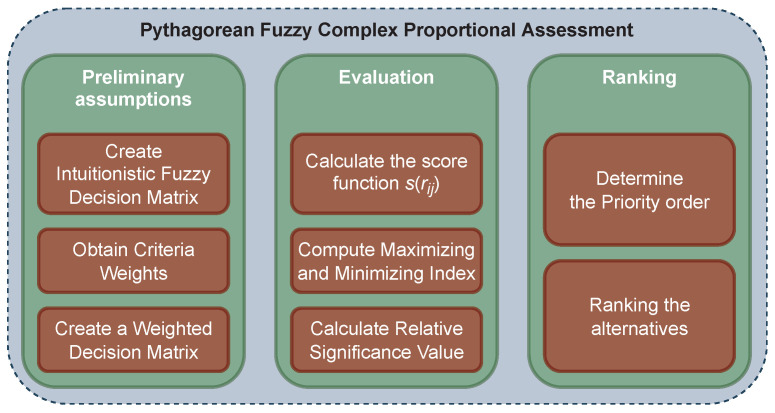
Flowchart of COPRAS method.

After determining the criterion weights and the Pythagorean fuzzy decision matrix, the COPRAS method will evaluate the various alternatives. The flowchart for this method is shown in Figure 2, and its steps are part of the whole process and as follows:

**Step VII:** Calculate the weighted decision matrix $D={\left[d_{ij}\right]}_{m\times r}$ where $d_{ij}=w_{j}n_{ij}=\left(\sqrt{1-{\left(1-\mu_{ij}^{2}\right)}^{w_{u}}},\;\vartheta_{ij}^{w_{j}}\right)$ where (j=1,2,⋯,r)

**Step VIII:** Calculate the score function

(12) $$h\left(d_{ij}\right)=\mu_{ij}^{2}-\vartheta_{ij}^{2}$$

where i=1,2,⋯,m; j=1,2,⋯,r.

**Step IX:** Determine the maximizing and minimizing index (13) and (14):

(13) $$h\left(U_{i}\right)=\frac{1}{\left|B\right|}\sum_{j\in B}h\left(d_{ij}\right)$$

and

(14) $$h\left(Y_{i}\right)=\frac{1}{\left|NB\right|}\sum_{j\in NB}h\left(d_{ij}\right)$$

where *B* is the set of benefit criteria and NB is the set of non-benefit criteria, for all (i=1,2,⋯,m).

**Step X:** Determine the relative significance value of each alternative (15):

(15) $$S_{i}=g\left(U_{i}\right)+\frac{\sum_{i=1}^{m}e^{g\left(Y_{i}\right)}}{e^{g\left(Y_{i}\right)}\sum_{i=1}^{m}\frac{1}{e^{g\left(Y_{i}\right)}}}$$

where i=1,2,⋯,m.

**Step XI:** Determine the priority order (16):

(16) $$T_{i}=\frac{S_{i}}{max{\;S}_{i}}\times 100$$

where i=1,2,⋯,m.

**Step XII:** Ranking of the alternatives:

The ranking of the alternatives is regulated in declining order based on the values of priority order. The highest final value has the highest rank.

By using the Table 10 and Table 11, the evaluations of the alternatives for the proposed approach are presented. For example, alternative $A_{4}$ was designated as the best choice and received a very similar rating to alternative $A_{5}$. In contrast, the worst rating was given to alternative $A_{2}$, ranked last.

### 4.2. Sensitivity Analysis

In this section, studies on the proposed approach’s sensitivity analysis have been conducted. First, the effect of the alternatives on the values of the obtained weights was investigated. For this purpose, five datasets were created in which one of the possible alternatives $A_{1}$–$A_{5}$ was not considered. Then, the criteria weights were determined for all datasets and compared with those obtained for the complete set of alternatives.

Table 12 shows the results obtained for global and local weights when excluding individual alternatives $A_{1}$–$A_{5}$ from the main decision matrix. For criterion $C_{1}$, the largest differences in weights are found for sets without alternatives $A_{3}$ and $A_{4}$. In contrast, the smallest difference between the weight values for criterion $C_{1}$ occurs for the set without alternative $A_{2}$. Concerning criterion $C_{2}$ and its weight reference value, the largest apparent difference in values was obtained for the sets with the exclusion of alternatives $A_{2}$ and $A_{5}$. In contrast, the smallest difference was obtained for the set with the exclusion of alternative $A_{5}$. In the case of criterion $C_{3}$, the smallest difference between the value of the global weight and the local weight occurred for the set with the exclusion of alternative $A_{5}$. In contrast, the largest difference occurred for the set with the exclusion of alternative $A_{1}$. For criterion $C_{4}$, the largest difference in weights occurred for the set without alternative $A_{1}$. In contrast, the slightest difference between the weight values for criterion $C_{4}$ occurs for the set without alternative $A_{4}$. Concerning criterion $C_{5}$ and its weight reference, the most apparent differences in values were obtained for the set with the exclusion of alternative $A_{2}$, and the smallest for the set with the exclusion of alternative $A_{5}$. For criterion $C_{6}$, the slightest difference between the value of the global weight and the local weight occurred for the set with the exclusion of alternative $A_{2}$. On the other hand, the enormous difference occurred for the set with the exclusion of alternative $A_{5}$. Concerning the criterion $C_{7}$ and its weight reference value, the most significant apparent difference in values was obtained for the set with the exclusion of alternative $A_{4}$. In comparison, a minor difference was obtained for the set with the exclusion of alternative $A_{1}$. For criterion $C_{8}$, the least difference between the value of the global weight and the local weight occurred for the set with the exclusion of alternative $A_{1}$. On the other hand, the most significant difference occurred for the set with the exclusion of alternative $A_{2}$. For criterion $C_{9}$, the most considerable difference in weights occurred for the set without alternative $A_{2}$. In contrast, the almost negligible difference between the weight values for criterion $C_{9}$ occurs for the set without alternative $A_{3}$. For criterion $C_{10}$, a minor difference between the global weight value and the local weight value occurred for the sets with the exclusion of alternatives $A_{2}$ and $A_{5}$. On the other hand, a tremendous difference occurred for the set with the exclusion of alternative $A_{1}$. To the criterion $C_{11}$ and its weight reference value, the most significant apparent differences in values were obtained for the set with the exclusion of alternative $A_{3}$, and the smallest for the exclusion of alternative $A_{4}$.

**Figure 3:**
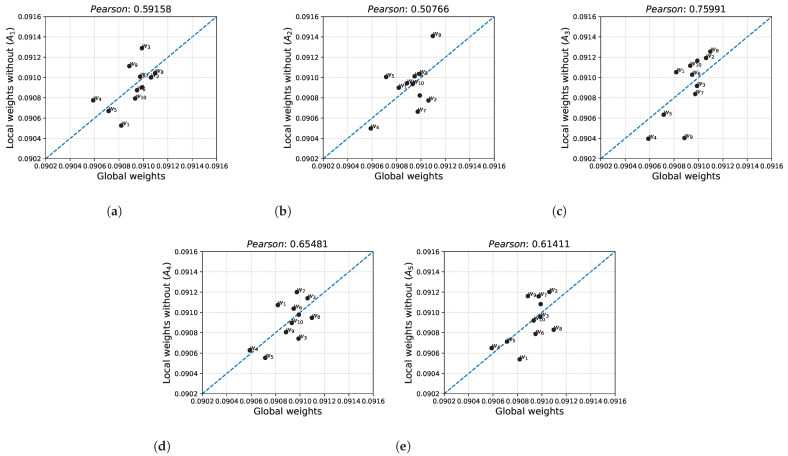
Global weights relative to local weights formed on the sets at the inclusions of alternatives $A_{1}$–$A_{5}$. (**a**) Without the alternative $A_{1}$; (**b**) Without the alternative $A_{2}$; (**c**) Without the alternative $A_{3}$; (**d**) Without the alternative $A_{4}$; (**e**) Without the alternative $A_{5}$ (Blue line means y=x).

Figure 3 plots the comparison of the weights obtained excluding the given alternative (local weights) against the weights obtained for the full dataset (global weights). Figure 3a plots the local weights for the set of alternatives without alternative $A_{1}$ against the global weights. The largest differences between the values are found for weights $w_{1},w_{3},w_{4}$ and $w_{9}$. Pearson’s correlation coefficient was 0.59158, indicating a minimal linear relationship between the considered weights. The relationship between the local weights for the decision matrix excluding the alternative $A_{2}$ and global weights is shown in Figure 3b. The lowest correlation was obtained from the cases considered for the comparisons obtained. For this comparison, the largest differences were observed for weights $w_{2},w_{5},w_{7}$ and $w_{8}$. Figure 3c shows the local weights for the set of alternatives without alternative $A_{3}$ relative to the global weights. The largest corollary was obtained for this comparison, where the Pearson coefficient was 0.75991. The largest difference between weight values was obtained for weight $w_{9}$. The correlation between the local weights for the decision matrix excluding alternative $A_{4}$ and the global weights is shown in Figure 3d. The resulting Pearson correlation coefficient value was 0.65481, indicating a low relationship between the studied weights. For this comparison, the largest value discrepancy occurred for the weight $w_{3}$. A final comparison of the local weights when excluding alternative $A_{5}$ with the global weights was presented in Figure 3e. The correlation between these weights, as with the other cases considered, is low. The largest differences between the values occurred for the weights $w_{1},w_{8}$ and $w_{9}$.

The next study related to the sensitivity analysis of the proposed approach was to change the criteria values by a threshold value of α. This study was designed to test how a minimal change in the criteria values affects the final evaluations of the alternatives. The PFN μ values were increased by a given threshold value, while a given threshold value decreased the PFN ϑ values. Small values of α parameters such as 0.001, 0.002, 0.005, and 0.01 did not demonstrate a significant effect, so it was decided to use a α parameter of 0.05. The evaluations of alternatives from the COPRAS method using this application of the approach for each criterion are presented in Table 13. No differences were observed in the evaluation of alternative $A_{2}$, which always obtained the last ranking. Although the evaluations of alternatives $A_{1}$ and $A_{4}$ changed with the threshold value for the set criteria, their positions were the same in all cases considered. The greatest differences were observed for alternatives $A_{3}$ and $A_{5}$. For the change in the value of the criteria $C_{1},C_{4},C_{5},C_{6},C_{8}$ by the given threshold value α, the alternative $A_{3}$ was ranked second, and the alternative $A_{5}$ was ranked 3rd. On the other hand, for changing the value of criteria $C_{2},C_{3},C_{7},C_{9},C_{10},C_{11}$ by a given threshold value α, alternative $A_{3}$ obtained the 3rd ranking and alternative $A_{5}$ obtained the 2nd ranking.

**Figure 4:**
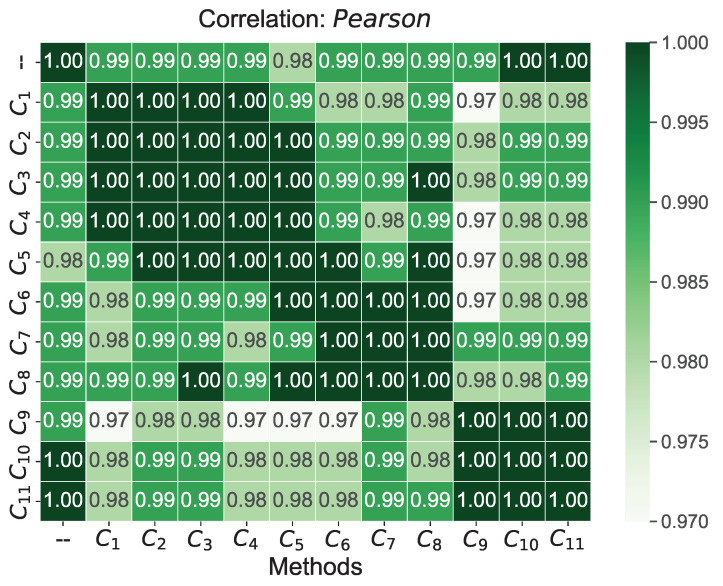
Pearson correlation coefficient values between scores obtained from modified $C_{1}$–$C_{11}$ criteria values by a threshold value of α=0.05 (darker colors mean a higher correlation value).

Using Figure 4, the Pearson’s coefficient values for the obtained scores from the COPRAS method when changing the threshold value α=0.05 for criteria $C_{1}$–$C_{11}$ are shown. The Pearson correlation values are in the range [1.00, 0.97], indicating highly similar scores from the given approaches. The smallest correlation values were obtained by the approach in which criterion a threshold value modified $C_{9}$. On the other hand, the highest correlation values were obtained by the approach in which a threshold value modified criterion $C_{11}$. The lowest correlation with reference scores was obtained with the approach where criterion $C_{5}$ was modified by a value of 0.05. On the other hand, the highest correlation with reference scores was obtained with the approaches where the threshold value α was included in criterion $C_{10}$ and $C_{11}$.

## 5. Conclusions

In this paper, we presented a new Pythagorean fuzzy entropy and its use in group decision-making. It was used to determine the relevance of criteria in an aggregated decision maker preference matrix. It was created because of the benefits of Pythagorean fuzzy sets on better modeling of uncertainty in data. Another aspect addressed in the paper was sensitivity analysis, which confirmed the validity and accuracy of the method used. The results confirmed this approach’s applicability in problems related to crop field evaluation using domain experts’ subjective opinions.

In the future, the presented Pythagorean fuzzy sets method can be used in additional applications. For example, the presented strategy can be used in future analyses by applying different fuzzy systems in decision matrices and solving various multi-criteria decision problems with unknown weights. Another research direction could be to use the proposed Pythagorean fuzzy entropy along with other MCDA approaches such as TOPSIS or VIKOR. Additionally, future research would need to address the aspect related to reference models of weights and their reflection by the proposed entropy.

## Figures and Tables

**Table 1:** Linguistic evaluation for rating criteria.

${\mathrm{DM}}_{i}$	$C_{1}$	$C_{2}$	$C_{3}$	$C_{4}$	$C_{5}$	$C_{6}$	$C_{7}$	$C_{8}$	$C_{9}$	$C_{10}$	$C_{11}$
$DM_{1}$	5	5	4	4	5	5	4	5	4	4	5
$DM_{2}$	3	3	5	5	3	3	5	4	5	4	3
$DM_{3}$	4	5	4	5	4	4	3	4	3	5	4
$DM_{4}$	5	4	5	3	4	5	4	3	4	4	5

**Table 2:** Linguistic terms for rating the importance of criteria and decision makers.

Linguistic Term	PFNs
Excellent—5	(0.96, 0.27)
Good—4	(0.83, 0.46)
Average—3	(0.65, 0.54)
Poor—2	(0.46, 0.73)
Very Poor—1	(0.38, 0.84)

**Table 3:** Linguistic evaluation for rating of the alternatives by decision makers.

${\mathrm{DM}}_{i}$	$A_{i}$	$C_{1}$	$C_{2}$	$C_{3}$	$C_{4}$	$C_{5}$	$C_{6}$	$C_{7}$	$C_{8}$	$C_{9}$	$C_{10}$	$C_{11}$
$DM_{1}$	$A_{1}$	6	7	6	5	6	7	6	7	6	7	7
	$A_{2}$	4	7	7	7	6	5	7	6	6	6	7
	$A_{3}$	7	5	7	6	7	6	7	5	7	5	5
	$A_{4}$	5	5	5	6	7	6	5	7	4	6	7
	$A_{5}$	7	5	5	4	6	7	4	7	3	6	6
$DM_{2}$	$A_{1}$	7	6	5	3	5	7	6	6	7	7	6
	$A_{2}$	6	7	6	4	4	7	7	4	5	4	5
	$A_{3}$	3	7	7	6	4	6	7	5	7	3	4
	$A_{4}$	3	5	7	5	5	5	4	5	7	3	6
	$A_{5}$	6	5	6	5	5	6	3	6	3	4	6
$DM_{3}$	$A_{1}$	6	6	7	7	6	6	4	6	4	6	7
	$A_{2}$	5	6	6	7	6	7	6	7	4	6	7
	$A_{3}$	5	6	5	7	6	7	5	5	5	7	7
	$A_{4}$	4	4	7	6	5	5	5	7	6	7	5
	$A_{5}$	7	3	7	4	7	5	7	3	6	5	4
$DM_{4}$	$A_{1}$	7	6	3	6	7	4	7	6	4	5	5
	$A_{2}$	7	6	4	5	4	4	6	3	7	7	6
	$A_{3}$	3	5	4	6	6	4	5	7	7	6	6
	$A_{4}$	4	7	6	5	6	6	6	5	6	6	4
	$A_{5}$	5	7	6	7	5	6	6	7	6	7	6

**Table 4:** Linguistic terms for rating alternatives.

Linguistic Term	PFNs
Excellent Good—7	(0.95, 0.27)
Very Good—6	(0.85, 0.44)
Good—5	(0.76, 0.63)
Medium Good—4	(0.65, 0.73)
Fair—3	(0.57, 0.79)
Medium Bad—2	(0.49, 0.87)
Bad—1	(0.41, 0.93)

**Table 5:** Decision Maker’s weights.

	${\mathrm{DM}}_{1}$	${\mathrm{DM}}_{2}$	${\mathrm{DM}}_{3}$	${\mathrm{DM}}_{4}$
Linguistic Term	5	4	3	5
Weight	0.28868	0.23832	0.18431	0.28868

**Table 6:** Aggregated Pythagorean fuzzy decision matrix.

$C_{i}$	$A_{1}$	$A_{2}$	$A_{3}$	$A_{4}$	$A_{5}$
$C_{1}$	(0.916, 0.340)	(0.852, 0.472)	(0.804, 0.556)	(0.672, 0.729)	(0.899, 0.387)
$C_{2}$	(0.891, 0.382)	(0.916, 0.340)	(0.851, 0.482)	(0.841, 0.507)	(0.836, 0.514)
$C_{3}$	(0.820, 0.519)	(0.864, 0.442)	(0.887, 0.421)	(0.894, 0.397)	(0.861, 0.446)
$C_{4}$	(0.825, 0.513)	(0.879, 0.437)	(0.878, 0.402)	(0.808, 0.532)	(0.824, 0.529)
$C_{5}$	(0.879, 0.416)	(0.769, 0.574)	(0.869, 0.431)	(0.869, 0.445)	(0.845, 0.486)
$C_{6}$	(0.896, 0.394)	(0.867, 0.459)	(0.847, 0.465)	(0.818, 0.512)	(0.882, 0.408)
$C_{7}$	(0.875, 0.419)	(0.916, 0.340)	(0.897, 0.403)	(0.772, 0.588)	(0.806, 0.535)
$C_{8}$	(0.891, 0.382)	(0.805, 0.537)	(0.850, 0.493)	(0.888, 0.422)	(0.906, 0.370)
$C_{9}$	(0.833, 0.498)	(0.860, 0.457)	(0.934, 0.316)	(0.856, 0.453)	(0.746, 0.599)
$C_{10}$	(0.905, 0.377)	(0.869, 0.431)	(0.825, 0.513)	(0.847, 0.462)	(0.858, 0.461)
$C_{11}$	(0.899, 0.387)	(0.902, 0.380)	(0.832, 0.503)	(0.852, 0.472)	(0.826, 0.483)

**Table 7:** Normalized aggregated Pythagorean fuzzy decision matrix.

$C_{i}$	$A_{1}$	$A_{2}$	$A_{3}$	$A_{4}$	$A_{5}$
$C_{1}$	(0.916, 0.340)	(0.852, 0.472)	(0.804, 0.556)	(0.672, 0.729)	(0.899, 0.387)
$C_{2}$	(0.891, 0.382)	(0.916, 0.340)	(0.851, 0.482)	(0.841, 0.507)	(0.836, 0.514)
$C_{3}$	(0.820, 0.519)	(0.864, 0.442)	(0.887, 0.421)	(0.894, 0.397)	(0.861, 0.446)
$C_{4}$	(0.513, 0.825)	(0.437, 0.879)	(0.402, 0.878)	(0.532, 0.808)	(0.529, 0.824)
$C_{5}$	(0.416, 0.879)	(0.574, 0.769)	(0.431, 0.869)	(0.445, 0.869)	(0.486, 0.845)
$C_{6}$	(0.394, 0.896)	(0.459, 0.867)	(0.465, 0.847)	(0.512, 0.818)	(0.408, 0.882)
$C_{7}$	(0.875, 0.419)	(0.916, 0.340)	(0.897, 0.403)	(0.772, 0.588)	(0.806, 0.535)
$C_{8}$	(0.891, 0.382)	(0.805, 0.537)	(0.850, 0.493)	(0.888, 0.422)	(0.906, 0.370)
$C_{9}$	(0.833, 0.498)	(0.860, 0.457)	(0.934, 0.316)	(0.856, 0.453)	(0.746, 0.599)
$C_{10}$	(0.905, 0.377)	(0.869, 0.431)	(0.825, 0.513)	(0.847, 0.462)	(0.858, 0.461)
$C_{11}$	(0.899, 0.387)	(0.902, 0.380)	(0.832, 0.503)	(0.852, 0.472)	(0.826, 0.483)

**Table 8:** Evaluation of entropy.

	$C_{1}$	$C_{2}$	$C_{3}$	$C_{4}$	$C_{5}$	$C_{6}$	$C_{7}$	$C_{8}$	$C_{9}$	$C_{10}$	$C_{11}$
Entropy	0.85575	0.83121	0.83869	0.87884	0.86614	0.84272	0.84011	0.82771	0.84901	0.84428	0.83842

**Table 9:** Evaluation of weights of criteria.

	$C_{1}$	$C_{2}$	$C_{3}$	$C_{4}$	$C_{5}$	$C_{6}$	$C_{7}$	$C_{8}$	$C_{9}$	$C_{10}$	$C_{11}$
Weight	0.08550	0.10005	0.09561	0.07181	0.07934	0.09322	0.09477	0.10212	0.08949	0.09230	0.09577

**Table 10:** The score function for alternatives.

$A_{i}$	$C_{1}$	$C_{2}$	$C_{3}$	$C_{4}$	$C_{5}$	$C_{6}$	$C_{7}$	$C_{8}$	$C_{9}$	$C_{10}$	$C_{11}$
$A_{1}$	−0.6869	−0.6786	−0.7809	−0.9510	−0.9647	−0.9641	−0.7195	−0.6726	−0.7821	−0.6892	−0.6874
$A_{2}$	−0.7747	−0.6388	−0.7324	−0.9665	−0.9279	−0.9519	−0.6561	−0.7795	−0.7557	−0.7343	−0.6822
$A_{3}$	−0.8194	−0.7432	−0.7101	−0.9689	−0.9617	−0.9470	−0.6984	−0.7427	−0.6454	−0.7840	−0.7699
$A_{4}$	−0.8973	−0.7572	−0.6957	−0.9462	−0.9606	−0.9353	−0.8219	−0.6917	−0.7564	−0.7570	−0.7494
$A_{5}$	−0.7184	−0.7621	−0.7356	−0.9492	−0.9524	−0.9600	−0.7935	−0.6551	−0.8421	−0.7510	−0.7659

**Table 11:** Scores and ranking of alternatives.

$A_{i}$	h (Ui)	h (Yi)	Si	Ti	Rank
$A_{1}$	−0.71219	−0.76306	−0.26231	102.47	IV
$A_{2}$	−0.71925	−0.79239	−0.25598	100	V
$A_{3}$	−0.73918	−0.76735	−0.28737	112.26	III
$A_{4}$	−0.76587	−0.79955	−0.29927	116.91	I
$A_{5}$	−0.75302	−0.78223	−0.29443	115.02	II

**Table 12:** Overview of the weights obtained for the decision matrix upon exclusion of individual alternatives $A_{1}$–$A_{5}$.

Weights	$C_{1}$	$C_{2}$	$C_{3}$	$C_{4}$	$C_{5}$	$C_{6}$	$C_{7}$	$C_{8}$	$C_{9}$	$C_{10}$	$C_{11}$
-	0.09082	0.09106	0.09099	0.09059	0.09072	0.09095	0.09097	0.09110	0.09089	0.09093	0.09099
$A_{1}$	0.09053	0.09100	0.09129	0.09078	0.09067	0.09087	0.09101	0.09104	0.09111	0.09079	0.09090
$A_{2}$	0.09090	0.09077	0.09104	0.09050	0.09101	0.09101	0.09066	0.09141	0.09094	0.09094	0.09082
$A_{3}$	0.09105	0.09119	0.09092	0.09040	0.09063	0.09103	0.09084	0.09126	0.09040	0.09112	0.09117
$A_{4}$	0.09107	0.09114	0.09074	0.09063	0.09055	0.09104	0.09120	0.09095	0.09081	0.09090	0.09098
$A_{5}$	0.09054	0.09120	0.09096	0.09065	0.09071	0.09079	0.09116	0.09083	0.09116	0.09092	0.09108

**Table 13:** Evaluations of alternatives $A_{1}$–$A_{5}$ from the COPRAS method for a threshold value of α=0.05 for criteria $C_{1}$–$C_{11}$.

$C_{i}$	$A_{1}$	$A_{2}$	$A_{3}$	$A_{4}$	$A_{5}$
$C_{1}$	100.57	100	113.63	118.44	113.59
$C_{2}$	101.79	100	116.46	121.66	116.65
$C_{3}$	102.53	100	116.37	121.45	117.17
$C_{4}$	101.48	100	116.94	120.50	116.37
$C_{5}$	103.67	100	119.59	123.14	118.23
$C_{6}$	105.23	100	120.15	123.04	119.70
$C_{7}$	107.57	100	122.57	128.01	124.13
$C_{8}$	105.80	100	122.46	126.96	122.05
$C_{9}$	106.53	100	118.20	127.93	124.23
$C_{10}$	105.25	100	119.85	129.51	125.47
$C_{11}$	105.72	100	123.23	133.01	129.19

## Data Availability

Not applicable.

## Abbreviations

The following abbreviations are used in this manuscript:

AHP	Analytical Hierarchy Process
ARAS	Additive Ratio ASsessment
BWM	Best-Worsst Method
COCOSO	Combined Compromise Solution
COMET	Characteristic Object METhod
COPRAS	Complex Proportional Assessment
CRITIC	CRiteria Importance Through Intercriteria Correlation
FUCOM	FUll COnsistency Method
IFS	Intuitionistic Fuzzy Sets
MABAC	Multi-Attributive Border Approximation area Comparison
MCDA	Multi-Criteria Decision-Analysis
MCDM	Multi-Criteria Decision Making
PFS	Pythagorean Fuzzy Set
PFN	Pythagorean Fuzzy Number
PROMETHEE	Preference Ranking Organization METHod for Enrichment of Evaluations
SPOTIS	Stable Preference Ordering Towards Ideal Solution
SWARA	Step-wise Weighting Assessment Ratio Analysis
VIKOR	VIseKriterijumska Optimizacija I Kompromisno Resenje
